# Antimicrobial drug resistant non-typhoidal *Salmonella enterica* in commercial poultry value chain in Chitwan, Nepal

**DOI:** 10.1186/s42522-020-00025-4

**Published:** 2020-10-01

**Authors:** Amy Nelson, Sulochana Manandhar, Juliana Ruzante, Arrogya Gywali, Bimala Dhakal, Santosh Dulal, Rupendra Chaulagai, Sameer M. Dixit

**Affiliations:** 1 RTI International, Research Triangle Park, NC USA; 2 Center for Molecular Dynamics Nepal (CMDN), Kathmandu, Nepal

**Keywords:** Poultry, Non-typhoidal *Salmonella serovars*, Nepal, Antimicrobial resistance, One health, Environment

## Abstract

**Background:**

Antimicrobial resistance (AMR) among bacterial pathogens is a fast-growing public health concern. AMR in non-typhoidal *Salmonella* serovars (NTS) among food animals is of special concern as this may transmit resistant pathogens to humans during handling or consumption of animal products. In Nepal, the possibility of AMR *Salmonella* serovars among food animals is an important area of research, particularly in light of the rapidly growing poultry industry, lack of surveillance and proper biosecurity measures; and paucity of relevant data. This study was conducted with the aim to estimate the burden of NTS and associated antimicrobial resistance in the environments of commercial poultry farms and the poultry carcasses in slaughter house. This study also intends to find some basic knowledge of the poultry farmers and their practice relating to the use of antimicrobials, vaccination and biosecurity measures.

**Methods:**

Taking one health approach, a cross-sectional study was carried out in Chitwan district of Nepal between May and October 2017. Various environmental samples viz. farm litter, feed, water, poultry faeces, vehicle swabs, farm swabs from 12 broiler poultry farms and various sections of poultry carcasses from 21 slaughter houses were aseptically collected. These were microbiologically assessed for the presence of NTS serovars and their phenotypic and genotypic indicators of antimicrobial resistance. The poultry farmers were also briefly interviewed regarding their basic biosecurity related knowledge and practices before collecting the environmental samples.

**Results:**

Overall, of total environmental samples collected, 50% (31/62) tested positive for NTS serovars with environmental swabs (70%, 8/12) being the most culture positive sample types. Similarly, of 159 tissue samples collected from 24 carcasses, 79% (126/159) were culture positive for NTS serovars. Nearly 97% (153/157) of isolates showed antimicrobial resistance to tetracycline, while 11% (17/157) to ciprofloxacin and 5% (8/157) of isolates were resistant against azithromycin. All 157 isolates were sensitive to meropenem. In terms of AMR genes, tetA (83%, 131/157), QrnS (40%,64/157), mefA (8%, 13/157) and VIM-1 (0.6%, 1/157) were detected in the isolates that corresponded to the AMR to tetracycline, ciprofloxacin, azithromycin and meropenem respectively. In farmers interview, only 42% (5/12) of farmers mentioned of using basic biosecurity measures such as applying lime powder around the farm; 84% (10/12) of farmers reported vaccinating their birds with some vaccine and 75% (9/12) of farmers used various antimicrobials prophylactically such as neomycin (33%, 4/12), colistin (33%, 4/12), furaltadone (33%, 4/12), doxycycline (25%, 3/12), sulfatrimethoprim (25%, 3/12) and tylosin (16%, 2/12).

**Conclusions:**

This study revealed gross contamination of farm environment and subsequent poultry meat samples with NTS serovars that were resistant to several clinically important antimicrobials. Further, inadequacy of even basic biosecurity measures and frequent prophylactic use of antimicrobials in the commercial poultry farms was observed. This reinforces an urgent need to raise awareness and implement proper biosecurity approaches from farms to slaughter houses in order to reduce the burden of NTS contamination of surrounding environment and poultry products. Further, high prevalence AMR among NTS isolates also underscores the need to strengthen the policies to prevent the rampant use of clinically used human antimicrobials in poultry sector.

## Background

In 2010, non-typhoidal *Salmonella enterica* (NTS) caused over 153 million illnesses and 122,000 deaths around the world, with children under 5 bearing a disproportionate burden [1, 2]. According to the World Health Organization (WHO) 2015, about 50% of those illnesses were caused by the consumption of contaminated food; however, animal contact, consumption of contaminated water were also potential vehicles [1, 2]. Of the food products linked to *Salmonella* contaminated, poultry is among the most frequent, resulting in international efforts to control and reduce the burden of *Salmonella* among poultry [3, 4].

In addition to the significant public health impact due to large burden of NTS illnesses, the increase in antimicrobial resistance (AMR) is a great concern [5]. Infections caused by drug resistant *Salmonella* tend to be more severe and lead to higher hospitalizations and deaths [6]. While global efforts are being targeted to monitor and track the development of drug resistant *Salmonella* globally [7, 8], systematic surveillance of illnesses and AMR can be extremely challenging in low resource countries and therefore the detection of pathogens and associated AMR may go undetected.

In Nepal, poultry production is a significant economic activity and represented 4% of the country’s gross domestic product in 2017 [9]. According to the Food and Agriculture Organization (FAO), in Nepal from 2007 to 2017, the production of poultry meat increased by 398% from 19.4 million chickens in 2007 to 75.4 million in 2017 [10]. And this number is expected to continue growing in the next coming years [11]. Various antimicrobials are commonly used in poultry production in Nepal to promote growth and prevent disease. Such unregulated consumption of antimicrobials is highly expected to increase in Nepal [12]. A 2012 survey conducted in Nepal found that the volume of sales of veterinary antimicrobials rose by over 50% between 2008 and 2012, and that more than 70% were obtained without a prescription [12]. The significant increase in production coupled with the likely misuse and overuse of antimicrobials in poultry production in Nepal might be accelerating the development of drug resistant *Salmonella* in the region.

Most surveillance efforts conducted in Nepal have focused on *Salmonella enterica* Typhi isolated from febrile humans [13, 14], and little knowledge exists concerning the burden and antimicrobial resistance of non-typhoidal *Salmonella enterica* in poultry sector of Nepal. Still, the limited data available highlight the data gaps that must be addressed. Therefore, we used a one-health approach to conduct a preliminary study on prevalence of non-typhoidal *Salmonella* and associated AMR in poultry sector in Chitwan region of Nepal using both phenotypic and molecular characterization methods.

## Methods

The study was conducted in the Chitwan district in southern Nepal between May and October 2017; this time period coincides with summer monsoons and typically expected to have the highest rates of human salmonellosis. Chitwan is one of the largest poultry egg and broiler producing districts in the country [15], with a population of 580,000 according to the 2011 census.

### Sample collection

#### Broiler farms

Twelve commercial poultry farms of Chitwan district registered with National Poultry Federation were selected to represent a range of production volume, housing types and hygiene. Upon receiving an informed consent from the farm owner, an informal interview was conducted by the research team with the farm owner to obtain information on volume of production and usual practices, such as the use of vaccines, antimicrobials, and hygiene and basic biosecurity measures. The GPS location of each farm was recorded using a mobile app. Environmental samples were collected by taking the following from each farm: approximately 20 g of farm litter; 25 g of poultry feed collected directly from the feed sack in use; and a spatula full of moist poultry feces from the chicken farm. Each sample was collected in a sterile container. Approximately 100 ml of drinking water used for poultry was collected in a sterile screw capped container by direct decanting. Further, a sterile cotton swab moistened with sterile skim milk was dragged along the inner walls of the poultry farm and immersed in vial containing 10 ml of sterile skimmed milk. A similar strategy was used with a sterile cotton swab to sample the wheels, side bars, and bird cages of the vehicle used for transporting birds from the farm to the slaughter facility. All samples were stored on ice and transported to the laboratory within 6 h.

#### Poultry tissue

Twenty-one slaughter houses in Chitwan district were selected such that they received the birds that were reared at the farms that were included and sampled in this study. The GPS points for each establishment were recorded using smart phone app. In each establishment, a chicken carcass was randomly selected. From each carcass, roughly 50 g of each seven different types of meat samples were collected using a sterile scalpel blade and were aseptically transferred into a sterile container. The sample types were gizzard, liver, heart, intestine, skin, spleen and muscle. These were stored and transported on ice to the laboratory within 6 hours of sample collection.

### Microbiological analysis

All samples except swabs were pre-enriched in Buffered peptone water (M614, Himedia laboratories, India) and incubated (STXBI45, Stericox Incubator, India) at 35 ± 2 °C for 18 h. Swab samples in skimmed milk were directly incubated (STXBI45, Stericox Incubator, India) at 35 ± 2 °C for 18 h. Samples were selectively enriched by transferring 100 μl of pre-enriched content into 9.9 ml of Rappaport Vassiliadis Broth (M880, Himedia laboratories, India) and incubated (STXBI45, Stericox Incubator, India) at 40 ± 2 °C for 18 h. A loop full of content was sub-cultured on Xylose Lysine Deoxycholate (XLD) agar (M031, Himedia laboratories, India) and incubated (STXBI45, Stericox Incubator, India) at 35 ± 2 °C for 18 h.

The inoculated XLD plates were examined for transparent red colonies with black centers, the characteristic feature of *Salmonellaenterica serovars.* For further confirmation of *Salmonella enterica* genus, the biochemical IMVic tests were performed for *Salmonella enterica* like isolated bacterial colonies. In IMViC biochemical tests, the isolates giving indole negative, methyl red positive, voges-proskauer negative, citrate utilization positive, alkaline slant by acidic butt with or without H_2_S in TSI (triple sugar iron) test, motile and urease negative results were confirmed as *Salmonella enterica* isolates. The typhoidal *Salmonella enterica* serovars viz., *Salmonella enterica* Typhi, Paratyphi A, B and C are highly adapted to humans and have no known animal or environmental reservoirs [16]. Thus, all *Salmonella enterica* serovars isolated from poultry and environmental samples in this study were assumed to be non-typhoidal *Salmonella enterica* serovars (NTS).

The antimicrobial susceptibility test was performed by modified Kirby Bauer disk diffusion method for azithromycin (15 μg), tetracycline (35 μg), ciprofloxacin (10 μg) and meropenem (10 μg). The disc diffusion breakpoint interpretation was carried out as per the CLSI (Clinical and Laboratory Standards Institute) 2017 guidelines [17]. The isolates were stored in glycerol stock at − 80 °C for future purpose.

### Molecular tests of antimicrobial resistance marker genes

The biochemically confirmed *Salmonella enterica* isolates were sub-cultured on XLD agar plate from the stored glycerol stock. Upon incubation, the plates were first examined for colony purity. Two to three well isolated colonies of *Salmonella enterica* were randomly selected from an overnight culture and re-suspended in 200 μl of sterile distilled water. Bacterial DNA was extracted using Quick-DNATM Fungal Bacterial Miniprep Kit (D6007, Zymo research, USA) following manufacturer instructions. Briefly, the bacterial cells in the suspension were physically disrupted by vigorously vortexing in BashingBead buffer (provided in the kit) followed by centrifugation (1730R, Gyrogen, Korea) at 10,000 x g for 1 min. The disrupted bacterial cells in the lysate were treated with Genome lysis buffer. The released crude bacterial DNA was collected on the Zymo-Spin™ IC Column followed by centrifugation (1730R, Gyrogen, Korea) at 10,000 x g for 1 min. Upon discarding the flow through, the DNA adsorbed on the column was washed twice with 200 μl of DNA Pre-Wash Buffer and 500 μl g-DNA Wash Buffer followed by centrifugation (1730R, Gyrogen, Korea) at 10,000 x g for 1 min. Finally, bacterial DNA was eluted from the column in 20 μl of DNA Elution Buffer and stored at 2–8 °C.

A Taq-man chemistry based real time PCR detection of antimicrobial resistance genes was carried out using a commercial kit Qiagen Microbial DNA qPCR Assay (330,025,Qiagen, Germany). The following antimicrobial resistance genes for specific antimicrobials were tested: QnrS (Assay catalog BPAR00441A, Qiagen, Germany) for fluoroquinolones; OXA-62 (Assay catalog BPAR00430A, Qiagen, Germany), NDM (Assay catalog BPAR00402A, Qiagen, Germany), IMP-1group (Assay catalog BPAR00398A, Qiagen, Germany) and VIM-1group (Assay catalog BPAR00403A, Qiagen, Germany) for beta-lactam antimicrobials; aacC1(Assay catalog BPAR00367A, Qiagen, Germany) for aminoglycosides; tetA (Assay catalog BPAR00449A, Qiagen, Germany) for tetracyclines; ermA (Assay catalog BPAR00442A, Qiagen, Germany) and mefA (Assay catalog BPAR00445A, Qiagen, Germany) for macrolides/lincosamides. A 25 μl of PCR reaction volume for each sample was set up containing 12.5 μl of hotstart qPCR mastermix, 9.5 μl of distilled water, 1 μl of qPCR assay constituting a mixture of AMR gene specific forward primer, reverse primer and specific hydrolysis probe and 2 μl of extracted DNA. In each batch of PCR run, a PCR positive control tube (added with the PCR positive control as provided by the manufacturer) and a no template control (NTC) tube (added with sterile distilled water) were included as internal controls. The PCR reaction for each AMR marker was prepared per sample. The real time detection of AMR genes was carried out in Sa Cycler-96 thermocycler (Sacace Biotechnologies, Italy) by using the following thermal-cycle protocol; initial activation at 95 °C/10 min and 40 cycles of denaturation at 95 °C/15 s followed by annealing, extension and detection (in FAM channel) at 60 °C/2 min. The ramp rate was set to 1 °C/second. After the completion of PCR program, the threshold cycle (CT) value for each well was calculated. The baseline and threshold were manually adjusted by normalizing the background signal from the NTC well. Finally, for data analysis, the threshold cycle values for all wells were exported to an Excel spreadsheet for scoring positive or negative results for each AMR marker for all samples.

### Statistical analysis

A statistical analysis was carried out using STATA software. The descriptive statistics of qualitative variables were expressed in absolute frequency (N) with percentage (%). That of quantitative variables was calculated in mean ± standard deviation (SD) or median with interquartile range (IQR). For the purposes of analysis in this study, the isolates showing intermediate antibiotic sensitivity were classified as sensitive.

## Results

Interviews were conducted with the farm owners from each of the 12 poultry farms visited. Broiler operations ranged in size from 70 birds to 1000 birds (median, 400; inter quartile range 200, 425). All farms used commercial pellet feed and raised birds of the same age in deep litter. At the end of each harvest, 75% (9/12) of the farms disposed of the farm litter through a contractor that is to be used as a fertilizer, while the rest disposed it by themselves in their own farms. Regarding employing biosecurity measures, approximately 42% (5/12) of farmers reported using lime powder around their farm, 16.6% (2/12) failed to maintain any biosecurity measures and remaining 41.6% (5/12) had no knowledge about biosecurity measures. Nearly 84% (10/12) of farmers reported vaccinating their birds with some vaccine. Seventy five percent (9/12) of farmers used antimicrobials, of which eight farms reported of obtaining the antibiotics from the feed supplier. The classes of antimicrobials used prophylactically in farms varied viz., neomycin (33%, 4/12), colistin (33%, 4/12), furaltadone (33%, 4/12), doxycycline (25%, 3/12), sulpha-trimethoprim (25%, 3/12) and tylosin (16%, 2/12).

### Prevalence of *Salmonella enterica* in the poultry setting

Twelve broiler farms were interviewed, but environmental samples could be collected from 11 farms only. A total of 62 environmental samples were collected from the farms, of which 50% (31/62) tested positive for NTS serovars. Almost 67% (8/12) of the farm and vehicle swab samples and 50% (6/12) of faecal samples were culture positive for NTS serovars. With exception of one farm, all farms had at least one *Salmonella enterica* culture positive sample. All collected poultry drinking water samples were culture negative. Table 1 shows the type of samples collected and their culture positivity rate for *Salmonella enterica* serovars.

**Table 1:** Percentage of *Salmonella enterica* culture positivity by sample types

Poultry tissue samples	N	Culture positive*,* n (%)	Environmental samples	N	Culture positive*,* n (%)
Muscle	24	20 (83%)	Farm swab	12	8 (67%)
Skin	24	19 (79%)	Feces	12	6 (50%)
Intestine	24	21 (88%)	Litter	12	4 (33%)
Gizzard	24	18 (75%)	Vehicle swab	12	8 (67%)
Heart	23	16 (70%)	Feed	12	5 (42%)
Liver	24	18 (75%)	Water	2	0 (0%)
Spleen	16	14 (88%)			
Total	159	126 (79%)	**Total**	62	31 (50%)
Total (tissue and environmental)				**221**	**157 (71%)**

In total, 159 poultry tissue samples including skin, muscle, heart, intestine, liver, gizzard, and spleen were collected from 24 chicken carcasses obtained from slaughter houses. Of these, 79% (126/159) were culture positive for NTS serovars. At least 70% of each tissue types were culture positive for NTS serovars; all chicken carcasses had at least four NTS culture positive samples (Table 1).

### Antimicrobial resistance profile

The antimicrobial susceptibility test results for 157 *Salmonella enterica* isolates from poultry tissue and environmental samples are shown in Table 2. Almost all isolates from environmental and tissue samples were resistant to tetracycline (35 μg). At least 10 and 5% of the isolates from both sample types were resistant to ciprofloxacin (10 μg) and azithromycin (15 μg), respectively. Resistance to meropenem (10 μg) was not detected in any of the isolates. Overall, the percentage of resistance to each antimicrobial was comparable between environmental and poultry tissue samples. Sample sizes were too small to measure the statistical difference in prevalence of AMR among various sample types.

**Table 2:** Phenotypic antimicrobial resistance results of *Salmonella enterica* isolates by sample types

Sample types	N	Tetracycline, n (%)	Ciprofloxacin, n (%)	Azithromycin, n (%)
Farm environmental samples	**31**	**29 (94%)**	**3 (10%)**	**2 (6%)**
Farm swabs	8	6 (75%)	0	0
Feces	6	6 (100%)	0	2 (33%)
Litter	4	4 (100%)	0	0
Vehicle swabs	8	8 (100%)	1 (13%)	0
Feed	5	5 (100%)	2 (40%)	0
Poultry tissue samples	**126**	**124 (98%)**	**14 (11%)**	**6 (5%)**
Muscle	20	20 (100%)	3 (15%)	1 (5%)
Skin	19	19 (100%)	3 (16%)	1 (5%)
Intestine	21	21 (100%)	0	1 (5%)
Gizzard	18	18 (100%)	4 (22%)	1 (6%)
Heart	16	16 (100%)	4 (25%)	0
Liver	18	16 (89%)	0	1 (6%)
Spleen	14	14 (100%)	0	1 (7%)
Grand total	**157**	**153 (97%)**	**17 (9%)**	**8 (4%)**

*All isolates were susceptible to meropenem

The PCR results for the markers associated with resistance to specific antimicrobials are shown in Table 3. The *tetA* gene, a marker for resistance against tetracycline, was the most prevalent, with over 80% of isolates from both the samples types testing positive. *QnrS* and *mefA* were next more common AMR markers in NTS isolates from both sample types. Out of the 10 farms, four had one or more isolates with only *tetA* detected, and no other markers. The isolates from five farms tested positive for co-existence of *QnrS* or *mefA* along with *tetA*. One of the farms had the isolates that were positive for all three markers: *tetA*, *QnrS* and *mefA*.. The AMR genes *aacC1*, *NDM, OXA-62, IMP-1* were not detected in any of tested NTS isolates.

**Table 3:** Occurrence and patterns of antibiotic resistance molecular markers detected by RT-PCR among *Salmonella enterica* isolates from poultry tissue (P) and environmental samples (E)

Genetic marker	Environment samples *N* = 31	Poultry tissue samples *N* = 126	Isolates with *only* this marker
*tetA*	25 (81%)	106 (84%)	12 (39%, E); 49 (39%, P)
*QnrS*	11 (35%)	53 (42%)	0 (E); 1 (0.1%, P)
*mefA*	3 (10%)	10 (8%)	0(E); 2 (0.2%, P)
*VIM-1*	0	1 (0.1%)	–
Both *tetA*and*QnrS*	10 (32%)	48 (38%)	
Both *tetA*and *mefA*	2 (6%)	4 (0.3%)	
Both *tetA*and *VIM-1*	0	1 (0.1%)	
Both *tetA, QnrS,* and *mefA*	1 (3%)	4 (0.3%)	

*None of the isolates were positive for the AMR markers *aacC1, NDM, OXA-62, IMP-1*

## Discussion

This is the first study, to our knowledge that has collected a range of environmental samples from broiler farms in Nepal with the aim to assess AMR in *Salmonella enterica*. The study findings revealed higher levels of *Salmonella* contamination and antimicrobial resistance than previously observed in poultry meat in Nepal. In interview with farm owners, it was found that only few broiler farmers implemented biosecurity measures. While Nepal regulates the use of antibiotics among food animals, an enforcement is not generally maintained.

A high prevalence of NTS was detected in both poultry tissue (79%, 126/159) and environmental samples (50%, 31/62). The gastrointestinal tract of poultry is one of most common natural niche for several serovars of NTS. The high prevalence of NTS in poultry carcasses indicated possible high level of visceral contamination of the meat products. One study conducted in retail meat shops of Kathmandu reported the detection of *Salmonella* in 14.5% of the chicken meat samples. A recent study conducted in 38 raw chicken meat samples collected from slaughter houses of Bharatpur, Chitwan reported that 26.2% of isolated organisms were *Salmonella* spp. Further, high prevalence of NTS in the environmental samples of the poultry farm suggested equally high levels of fecal contamination of the surrounding suggesting lack of proper bio-security measures.

Similar studies conducted in other low- and middle-income Asian countries have reported a wide range of prevalence of NTS in poultry and environmental samples. Studies in India found a range of 0 to 10% NTS prevalence among poultry meat and tissue [18, 19]. Another study on NTS detected 0.6, 1.7, and 7.4% of NTS in poultry feces, drag swabs, and feed samples, respectively [19]. In Vietnam, NTS prevalence among broiler farms was found to be 45.6% [20] in one study and 64.7% in another [21]. A study of poultry meat carcasses in Yangon, Myanmar during 2014–2015 found a 98% prevalence of *Salmonella* among 141 retail markets [22].

Among *Salmonella* isolates from the previous study of retail meat in Chitwan, 85% (*n* = 23) were multi-drug resistant. The most common antibiotics for *Salmonella* resistance were ampicillin, nitrofurantoin, and doxycycline. Similar to our findings, the Vietnamese studies detected high percentages of NTS isolates resistant to tetracycline varying from 40% [20] to 78% [21] and low resistance to ciprofloxacin. Other studies of poultry and poultry farms from India found 100% of the NTS isolates to be tetracycline resistant [18, 19]; and one of the studies reported a routine use of oxytetracycline as a feed additive. In Myanmar, 54 and 9% of NTS isolates from retail chicken samples were resistant to tetracycline and ciprofloxacin, respectively [22].

In this study, the *QnrS* gene was detected in 16% of samples overall. However, none of the isolates that were resistant to ciprofloxacin had the *QnrS* marker; the majority specimens with the marker tested sensitive to ciprofloxacin indicating possible alternative genetic mechanism for observed ciprofloxacin resistance. The burden of antimicrobial resistance genes in bacterial isolates may depend on the extent of specific antibiotic agent used in that setting. The prolong use of antibiotics, particularly those of broad spectrum, may exert a selective pressure on the microbial community in the setting, which may facilitate rapid emergence and selection of antimicrobial resistant isolates and increased horizontal transmission of antimicrobial resistance genes. As antibiotics are extensively used in poultry in an unregulated way, mostly in developing countries, AMR among poultry should be closely monitored by the surveillance of AMR genes in poultry isolates.

One of the limitations of this study is the lack of bacterial serotyping method for specific identification of NTS serotypes. The selected bacterial isolates were considered to be one of the *Salmonella enterica* serotypes based on the use of *Salmonella* specific sample enrichment method, the colony characteristics on selective media and biochemical characteristics. Further, it was assumed that all *Salmonella enterica* serovars isolated in this study were non-typhoidal *Salmonella serovars* based on the fact that the typhoidal *Salmonella* serovars have only the humans as reservoirs. However, this study cannot guarantee that all *Salmonella enterica* isolates from samples were NTS. For example, it could not be ruled out if there were any prior human contamination of the samples, such as resulting from the handling of poultry samples by a person infected or colonized with typhoidal *Salmonella* serovars (*Salmonella enterica* Typhi and Paratyphi).

However, the main aim of this study was to assess the burden of AMR *Salmonella* in poultry meat samples that were meant for human consumption and in the surrounding where the commercial chicken are raised. Such findings are important to reinforce and educate about biosecurity measures to reduce the burden of *Salmonella* contamination of animal derived food.

## Conclusions

In this study, a high prevalence of antimicrobial resistance NTS was observed in the poultry samples and in the farm environments. Further, a high prevalence of AMR NTS isolates was observed in specimens taken from transporting vehicles which could implicate in an easy spread of resistant NTS beyond farm. Additionally, low levels of knowledge and practice regarding biosecurity amongst farmers found this study could further exacerbate the existing problem of high burden of antimicrobial resistant NTS in poultry industry. The findings from this study thus emphasizes on the need to educate and enforce optimal biosecurity measures at all levels of food supply value chain from the farms, slaughter houses to the retail shops so as to reduce the burden of *Salmonella* contamination of animal derived foods. Additionally, this study emphasises on the need to conduct a routine surveillance of AMR on potential human pathogens contaminating the human foods of animal origin so as to assess the burden and diversity of AMR genes that may have direct impact on human health. In this study, it was found that the use of antibiotics as prophylactics in poultry was a common finding. Given a high burden of AMR genes among NTS isolates in this study, it also underscores the urgency of proper regulation of antimicrobials in poultry industry to prevent an inappropriate use, particularly of last resort clinical antimicrobials like colistin. Additionally, awareness programs at local farm and community levels on antimicrobial stewardship and impact of poultry linked AMR on human health may be more effective and sustainable to prevent the misuse of antimicrobials in poultry sector.

## Data Availability

The datasets during and/or analysed during the current study will be made available from the corresponding author on request.

## Abbreviations

AMR: Antimicrobial Resistance
CLSI: Clinical Laboratory Standards Institute
FAO: Food and Agriculture Organization
NTS: Non typhoidal *Salmonella enterica*
WHO: World Health Organization
XLD: Xylose Lysine Deoxycholate agar
